# Perceptions and acceptability of some stakeholders about the bovine tuberculosis surveillance system for wildlife (Sylvatub) in France

**DOI:** 10.1371/journal.pone.0194447

**Published:** 2018-03-15

**Authors:** Julie Rivière, Yann Le Strat, Pascal Hendrikx, Barbara Dufour

**Affiliations:** 1 USC EPIMAI Unit, Anses, Ecole Nationale Vétérinaire d’Alfort, Maisons-Alfort, France; 2 Santé publique France, French National Public Health Agency, Saint-Maurice, France; 3 Surveillance Coordination and Support Unit, Agence Nationale de Sécurité Alimentaire Nationale, French Agency for Food, Environmental and Occupational Health and Safety (Anses), Maisons-Alfort, France; Universidade Nova de Lisboa Instituto de Higiene e Medicina Tropical, PORTUGAL

## Abstract

Bovine tuberculosis (bTB) is a common disease of cattle and wildlife, with economic repercussions and implications for animal and human health. The surveillance of bTB in wildlife is particularly important, to shed light on the epidemiological role of wild species and for the adaptation of control measures. In France, a bTB surveillance system for free-ranging wildlife, the Sylvatub system, was launched in 2011 on wild boars, red deer, roe deer and badgers. It relies on active and passive surveillance activities, constrained by practical difficulties, such as the accessibility of wild animals, and regulatory rules for the trapping of badgers, for example. We report here the first assessment of stakeholders’ perceptions of the Sylvatub system and its acceptability, based on 20 individual semi-structured interviews with three types of stakeholder (collectors, coordinators, officers) in areas with different rates of bTB infection. With the caveat that these findings cannot be assumed to be representative of the national situation, we found that the Sylvatub system was considered useful by all the stakeholders interviewed. Those from the world of hunting participate in surveillance mostly to help livestock farmers, who are not systematically involved in bTB surveillance in wildlife. Many practical and regulatory constraints were raised, which could be offset by recognition of the work done by the “hunting community”, to maintain the willingness of these individuals to participate. We also identified a need for improvements in communication and information. Qualitative information, such as that collected here, is essential to improve our understanding of the reasons favoring and disfavoring participation in surveillance, and should be taken into account in the evaluation process. These results are relevant to hunters and to veterinary authorities wishing to identify the determinants of participation in the Sylvatub system. They could provide support for decision-making processes to improve surveillance strategies.

## Introduction

Bovine tuberculosis (bTB), which is caused principally by *Mycobacterium bovis*, is a chronic disease affecting livestock, companion animals, wild species and humans [1]. It is a challenging human and animal health problem that jeopardizes the economic sustainability of the livestock sector (due to surveillance costs, movement restrictions, compensation for slaughtered cattle) and wildlife conservation [2]. According to the European Commission, France has officially been considered bTB-free since 2000 (Commission Decision 2003/467/EC, lastly amended by the Commission Implementing Decision 2016/168 of 5 February 2016), as this status is obtained if the percentage of bovine herds confirmed as infected with tuberculosis has not exceeded 0,1% per year of all herds for six consecutive years–this status does not depend on bTB cases in wildlife (Council Directive 64/432/33C of 26 June 1964). However, a few infected herds and cases in wildlife (red deer, *Cervus elaphus;* roe deer, *Capreolus capreolus;* Eurasian wild boar, *Sus scrofa;* and badgers, *Meles meles*) are detected in certain areas each year since a decade. Eradication of the disease requires an understanding of the role of wild animals in bTB epidemiology [3, 4, 5]. A national surveillance system for bTB in wildlife, the Sylvatub system, was therefore launched in France in 2011. This system consists of three independent surveillance system components (SSCs) applied according to a level of bTB risk: passive surveillance by carcass examination during hunting, passive surveillance of animals found dead or dying, and targeted, risk-based investigations in areas of medium and high risk.

Regular evaluations of surveillance systems are crucial, to increase their sustainability, effectiveness and efficiency [6, 7]. The effectiveness and cost-effectiveness of the Sylvatub system have already been assessed with a quantitative scenario tree model [8, 9]. This method represents the decision-making process and the choices made by the stakeholders (declaration of suspected infections, collection of dead animals, etc.) as the nodes of a tree. However, it is difficult to evaluate the behavior of individuals and their motivations. This can depend on many parameters, some specific to the individual (health awareness), or the environment (type of hunting, for example) and may affect surveillance functioning [10].

According to Brugere *et al*. (2017) [11], two main drivers influence the ability to detect and report disease and the willingness to participate in surveillance activities: technological and behavioral factors, at the individual or institutional scale. Willingness to declare suspected infections in passive surveillance programs is influenced by the acceptability of the program to those in the field, in terms of its operational, and economic aspects. The operational aspects are represented by the complexity of the system and the operational consequences of a suspicion or a confirmation case, such as increased surveillance; and the economic aspects are for example the impact of the cost of surveillance activities on the motivation of stakeholders involved, or the financial compensation that could be given to some of them. Behavioral effects, such as the hunters’ awareness of the disease, their willingness to report suspected infections, and their acceptance of the surveillance and control measures, are thus important and should be included in evaluations of the performance of the system, and in decision-making processes [10]. Furthermore, an understanding of the factors driving participation in a surveillance system is essential, to maintain the awareness and operational involvement of all stakeholders, thereby increasing the sensitivity of case detection in the long term.

For the Sylvatub system, semi-quantitative evaluation with the OASIS method highlighted a limited acceptability of surveillance activities, due to practical constraints and negative consequences of the declaration of suspected cases [12]. Furthermore, our quantitative scenario tree model revealed differences between the number of theoretical suspected cases we would expect to be recorded by stakeholders according to our model, and the real number of suspected cases declared. This difference may be reflect inadequate parameterization of the model, or an operational dysfunction of surveillance in the field, in relation to a lack of stakeholder awareness (leading to suspected cases of infection being missed), a lack of communication between actors or fears about negative consequences of reporting suspected infections (leading to underreporting) and resulting in poor acceptability. Moreover, although we cannot rule out defects in the input parameters of our model, the number of declared cases of suspected infection remains low, given that the presence of any abscess should be considered suspect in carcass surveillance, according to the regulations of the Sylvatub system.

Thus, the evaluation of a surveillance system cannot be based solely on its ability to detect cases (effectiveness) and on its cost, as these attributes may be influenced by the acceptability of the measures by stakeholders [13]. Evaluations must therefore also consider the stakeholders’ perceptions of the system, particularly if practical difficulties are encountered in the collection of animals, for example, due to the need for voluntary participation (which may or may not be recompensed), as in the Sylvatub system. The multi-partner nature of the Sylvatub system also renders its organization complex, and relationships between stakeholders could influence their participation. It was not possible to evaluate social and behavioral factors (awareness, acceptability, communication) with the quantitative scenario tree modeling method. We therefore investigated other complementary methods for this evaluation.

The objectives of this study were to perform the first qualitative evaluation of the sustainability of the Sylvatub system in the medium and long term, by (i) investigating local perceptions of utility, concerns and acceptability, for the whole system and for each SSC, (ii) identifying key factors influencing the stakeholders’ willingness to participate (incentives and disincentives) and (iii) investigating the relationship between stakeholders. Individual semi-structured interviews were conducted with each type of stakeholder and for each level of risk.

## Materials and methods

### Ethics statement

This study involved no medical research on human or animal and was based solely on qualitative interview-based research. No ethics committee approval was therefore required for this study. General ethical principles were respected: before each interview, the investigator presented the study objectives and asked for verbal informed consent from all participants; after the interviews, all data were rendered anonymous, to respect the privacy of participants.

### Description of the Sylvatub system and of the network of participating stakeholders

**Fig 1:**
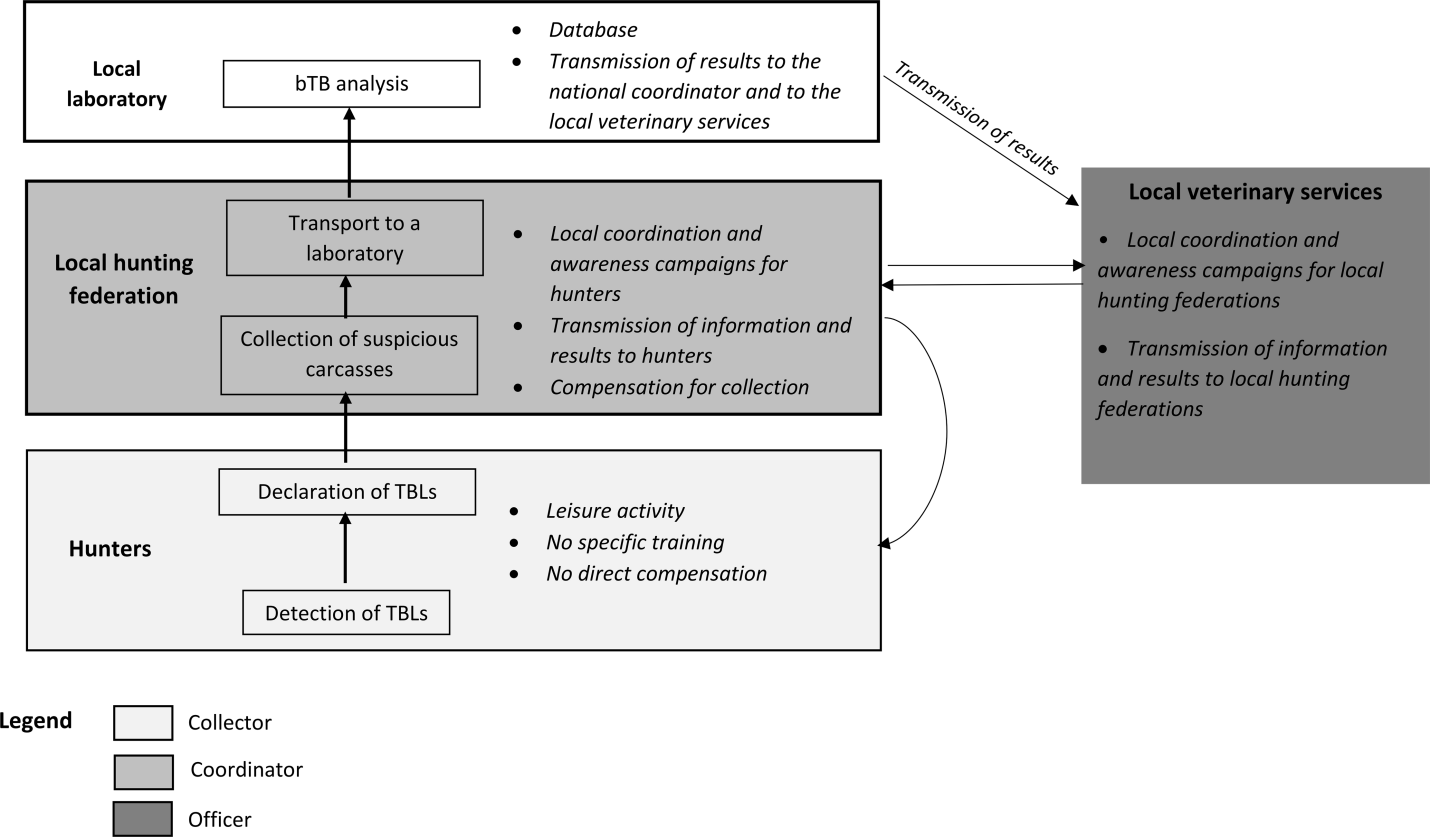
Schematic view of the functioning of the EC-SSC and of the network of stakeholders involved.

**Fig 2:**
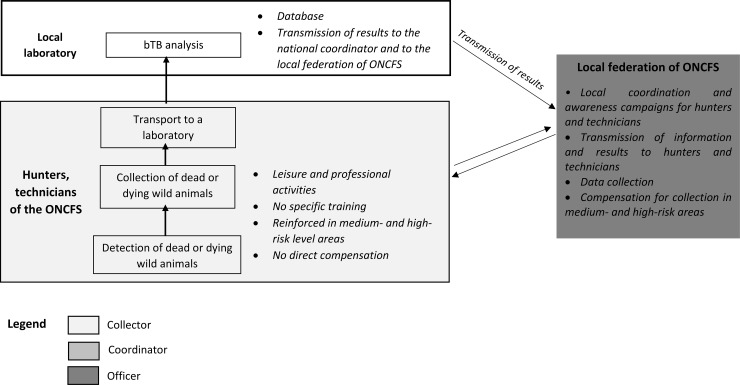
Schematic view of the functioning and network of participants for the SAGIR-SSC.

**Fig 3:**
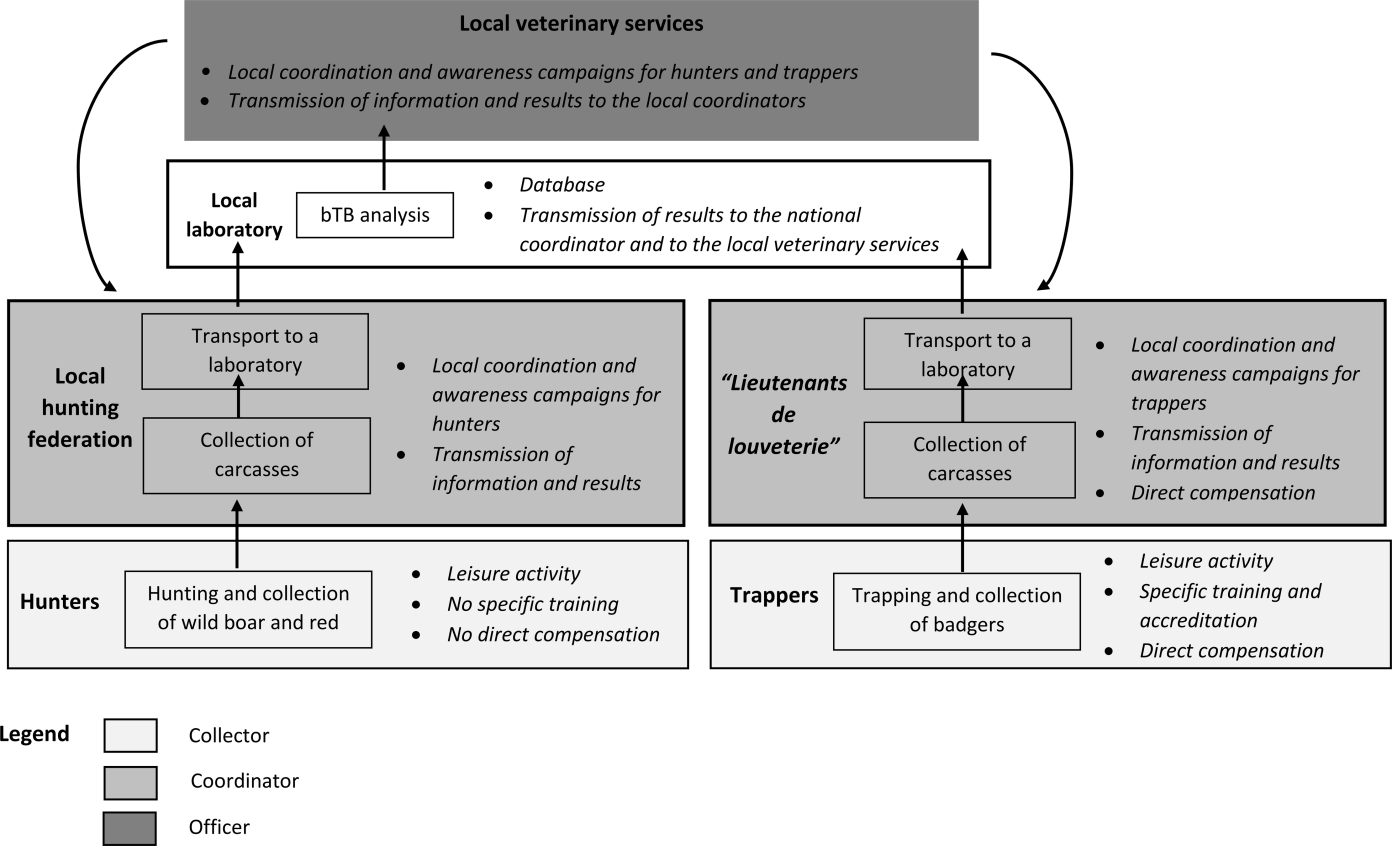
Schematic view of the functioning and network of stakeholders for the PSURV-SSC.

The Sylvatub system consists of three SSCs applied according to geographic risk, which is determined as a function of outbreaks in cattle and wildlife. Three levels of risk have been defined (low, medium, and high), with regular evaluation according to changes in the number of cases identified in cattle and wildlife. The three SSCs have been described elsewhere [8] and correspond to a combination of passive (reporting of suspect cases by stakeholders) and active (collection of samples according to a predetermined sampling) surveillance activities:

1. EC-SSC (Fig 1): passive surveillance based on the examination of carcasses of wild boar, red deer and roe deer killed by hunters, applied in all areas (*i*.*e*. regardless of local risk). This component involves the post mortem examination of the carcass by the hunter, and the voluntary submission of carcasses with macroscopic tuberculosis-like lesions (TBLs). Hunters are asked to submit any game animals with TBLs to laboratories for testing, free of charge, and the local hunters’ federation is reimbursed for carcass management (sampling and transport). The hunter’s awareness of TBLs is crucial for their detection, and the acceptability of the process is vital for the reporting of suspected cases. Some hunters receive specific training in the recognition of abnormal carcasses, but such training is not obligatory.
2. SAGIR-SSC (Fig 2): passive surveillance on dead or dying animals, for wild boar, red deer, roe deer and badger. This component relies on a preexisting network, the SAGIR network, in which stakeholders in the field (hunters, local hunting federations and technicians from the National Hunting and Wildlife Office, the ONCFS) provide an inventory of dead or moribund animals found in forests or at the roadside. They transport the animals concerned to a laboratory for investigations at their own expense, through their local federations (the analysis depends on the results of the necropsy) [14]. Within the Sylvatub system, the SAGIR network receives assistance, free of charge, in areas of medium or high risk, which concern the collection of large animals and systematic bTB analysis, even in the absence of TBL detection on necropsy.
3. PSURV-SSC (Fig 3): planned active surveillance on hunted wild boars, red deer and trapped badgers. Systematic bTB analysis is conducted on a sample of 15 badgers trapped within 1 km of bTB cattle outbreaks in medium-risk areas, and on samples of about 100 badgers and/or about 100 wild boars and/or about 60 red deer in larger areas within high-risk zones, according to the geographic distributions of these species (national regulatory text DGAL/SDSPA/2015-556). Animals are collected even if no macroscopic TBLs are detected by stakeholders. Wild boar and red deer are collected by hunters, under the supervision of the local hunting federation, and badgers are collected by trappers (specific training and accreditation required), under the supervision of “*lieutenants de louveterie*” (state employees responsible for wildlife management).

The Sylvatub system relies heavily on volunteers, some of whom are recompensed for their participation (such as trappers), whereas others receive no reimbursement for these activities carried out in the context of leisure activities (hunting). The effectiveness and sustainability of the Sylvatub system depend on all stakeholders, from collectors to officers, feeling concerned about bTB in wildlife. Furthermore, Sylvatub was set up only recently, in 2011, and involves different types of stakeholders with little previous experience of working together. The creation of the network of stakeholders required the positioning of each group with respect to the others. Given the fundamental role of hunters, trappers and their institutions in surveillance, a clear understanding of the impact of incentives, disincentives and external influences on their behavior and participation in surveillance activities is required [11].

### Data collection: Study design

#### Targeted population, geographic areas and period

Targeted population: We identified all the stakeholders involved in the Sylvatub system, and assigned them to three categories, according to their role in the surveillance system. The first category “collectors” corresponds to those working at the front line in the field, such as hunters for EC-SSC and PSURV-SSC, trappers for badgers in PSURV-SSC and the technicians of the SAGIR network for SAGIR-SSC. The second category “coordinators” corresponds to those coordinating surveillance activities: local federations of hunters, local federations of the ONCFS, “*lieutenants de louveterie*”. The third category “officers” corresponds to those representing the state, such as the local veterinary services.

Geographic areas: We took into account the level of risk, which may influence the experiences of each category of stakeholders, thereby affecting their perception of the utility of the Sylvatub system and its acceptability, by conducting this study in four different *départements* (French administrative areas essentially equivalent to counties). Due to financial and time constraints, we choose to investigate one department at low risk (*Gers*), one at medium risk (*Haute-Garonne*), and two at high risk (*Pyrénées-Atlantiques*, *Landes*); the later as bTB cases occurred in these areas in cattle and wildlife since several years, which could influence stakeholders’ perceptions, and as these two areas have similar stakeholders’ networks but different modes of operation.

Investigator and period: All stakeholders were interviewed by the same person, a Masters student (Management, Social and Human Science Masters), between May and July 2015.

#### Semi-structured interviews and themes

The number of interviews was determined by the variety of situations in the field: stakeholders, risk levels, SSCs. Participants were selected according to their role in surveillance, so as to represent all categories of stakeholders, but also according to their availability and willingness to participate. In qualitative approaches of this kind, sample quality is more important than sample size [7, 15], as the objective is to maximize the diversity of situations and, thus, of data collected as points of view. The ideal number of interviews is considered to have been reached when no new information is collected, which is called the theoretical saturation [16, 17]. Participants were first contacted by phone and informed about the study. Semi-structured interviews were conducted individually, face-to-face, at a location of the participant’s choice. This method has the advantage of being standardized with the use of guidelines for the interview developed by the authors (S1 File), but it is also flexible as participants were encouraged to provide any additional information they considered relevant, and can be used to collect both opinions and information about attitudes. Participants were informed that their opinions would remain anonymous and verbal consent was given at the start of the discussion.

We took into account the various aspects of the surveillance system relating to acceptability: objectives of the surveillance, functioning of the system, relationship between stakeholders, perception of their own utility and that of the other stakeholders, satisfaction with their relationship within the network, consequences of a suspicion for each stakeholder (changes in their activity) and on the relation between them, trust in the system and in other stakeholders involved in the system [7, 18]. Four main themes were discussed during the interviews: (i) a description of activities relating to wildlife and to the Sylvatub system, (ii) the specific factors acting as incentives and disincentives for participation in the Sylvatub system (internal or external to the Sylvatub system), (iii) the relationship between stakeholders and coordination activities, and (iv) the participants opinions concerning the utility of the Sylvatub system. These themes were defined on the basis of a preliminary exploratory interview with the national coordinator of the system, a national representative of the hunting federations and the regional coordinator for bTB in the South-West.

### Data analysis

The interviews were analyzed by transcribing the recordings by theme, according to the category of stakeholder, assessing the different feelings expressed and identifying any inconsistencies or contradictions. We analyzed the interviews by focusing on several dimensions:

- An operational (technical) dimension, considering the functioning of a recently implemented surveillance system;
- A social dimension, in the context of the creation of a new network of stakeholders, some of whom had never worked together before the creation of the Sylvatub system;
- An economic dimension, considering the perception of financial compensation for some stakeholders and whether such compensation is sufficient to ensure high levels of participation [19];
- A political dimension, as the Sylvatub system involves two Ministries, the Ministry of Agriculture and the Ministry of the Environment, which may have conflicting interests or viewpoints;
- A regulatory dimension, as certain surveillance activities, such as badger trapping, are subject to rules and regulations that the stakeholders may perceive as constraints.

The data presented in the results section reflect the anonymous observations and opinions expressed by respondents. To protect participant confidentiality, their identities, ages, sex, training and experience are not detailed in this paper, although the category of actor and the *department* are linked to citation. The verbatim citations have been translated from French. Only the category of stakeholder and the risk level of the *département* are indicated for each citation, to preserve the anonymity of the participants.

## Results

The number and characteristics of the stakeholders interviewed are presented in S1 Table. In total, 20 semi-structured interviews were performed in four *départements*, but some of the stakeholders had multiples roles. Thus, in total, 14 collectors (8 hunters, 1 trappers, 5 hunter/trappers), 9 local coordinators (4 from a local hunting federation and 5 *lieutenants de louveterie*), and 5 officers (3 representatives from local veterinary services and 2 from the ONCFS) were interviewed (only 3 persons refused to participate to this study, especially for time constraints). Interviews lasted between 2 and 3 hours on average.

### Knowledge of bTB in wildlife and its surveillance

Several training campaigns were run to provide stakeholders information about bTB, as they are not health professionals, but a number of participants, including some in high-risk areas, said that they lacked knowledge about bTB and its surveillance: “*I know that bTB is a disease*, *but that’s all*. *We are not specialists*, *and it’s difficult when farmers ask us for explanations*” (*lieutenant de louveterie*, high-risk). The epidemiological role of wild species was also unclear: “*Is it the cow that infected the badger or the other way round*? *I don’t know*” (farmer/hunter/trapper, high-risk).

### Determinants of the stakeholder participation (incentives)

- A feeling of utility and the need to help farmers: Stakeholder awareness seemed to depend heavily on the epidemiological situation regarding bTB. Indeed, in high-risk areas, wildlife surveillance was considered necessary (although not sufficient) for the control of bTB in cattle populations, by all categories of stakeholder. The main motivating factor was a feeling of utility, helping farmers: “*I do it to support the farmers I know*” (hunter/trapper, high-risk); “*We have to do something*. *Guys are trapping because they want to help farmers who are worried because they know there are cases of bTB in the area*” (hunters’ federation, high-risk).
- Surveillance for scientific knowledge: Several stakeholders highlighted the importance of better understanding the role of wild species in bTB epidemiology as one of the factors driving their participation in either passive or active surveillance: “*We thought we had to know*, *and that meant we had to look*, *and so we had to trap badgers”* (hunters’ federation, high-risk).
- A fascinating, fun, leisure activity: Most of the collectors and coordinators indicate that they made hunting or trapping as a leisure activity, as a passion. The constraints inherent to the Sylvatub system must therefore be few and weak, so as not to dissuade these stakeholders from taking part in surveillance: “*It should not be forgotten that hunting is initially a leisure activity*, *not a constraint*” (hunters’ federation, high-risk).
- Financial compensation, not essential but synonymous with recognition: Financial aspects were not seen as the principal motivation for participation. However, compensation for trapping activities is perceived as a form of recognition and appeared to be important for keeping trappers motivated in the long term: “*People don’t do it for the money*, *but it makes them feel good to know that their efforts have been recognized and that they have been rewarded*” (*lieutenant de louveterie*, high-risk). Some stakeholders even suggested replacing the financial compensation by more concrete recognition, in the form of material, for example. Some local coordinators agreed to take on some of the running costs at their own expense, as they simply wished to support farmers: “*We spent a lot of time there but we didn’t ask for compensation in the first two years*. *We felt that we were helping farmers by doing that*” (hunters’ federation, high-risk).

### The constraints and disincentives for participation

- Practical, economic and material constraints: A lack of resources has been reported for passive surveillance (EC-SSC and SAGIR-SSC), particularly in terms of the vehicles available, which acts as a practical constraint on animal collection and transport. However, some stakeholders have adapted their practical organization to facilitate collection activities, reflecting their willingness to be involved in the improvement of the Sylvatub system.
- Trapping badgers, a time-consuming activity: Several participants drew attention to the heavy workload involved in badger trapping, with the checking of traps every morning taking one to two hours. This aspect was also sometimes accompanied by feelings of failure, as trapping is a difficult activity for which success rates may be very low, which can be discouraging: “*When trappers do not catch anything*, *they are demotivated*, *discouraged*. *It’s understandable*, *because it takes time*” (*lieutenant de louveterie*, high-risk). These constraints make it more difficult to motivate trappers, and had consequences for the population of stakeholders active in trapping, most of whom are retire and, therefore, have more time at their disposal.
- Regulatory constraints: Several stakeholders mentioned the complexity of the working environment, converting leisure activities into highly regulated practices. This requires the stakeholders to update their knowledge continually, and some said that they felt overwhelmed by the regulatory and administrative aspects: “*I don’t know what to do*. *It's a whole load of skills and it keeps changing*. *There are more instructions*, *and if you want to do it right you have to follow them and keep up with the changes*. *It’s all a bit nerve-wracking*” (local federation of ONCFS, high-risk). Moreover, badger trapping requires an order from the prefecture, which entails administrative delays that are sometimes taken badly by the stakeholders, who feel that urgent action is required when bTB cases are picked up in a high-risk area.
- Relational constraints between actors: Relational constraints were mentioned by many stakeholders, sometimes operating within a stakeholder category: “*If we have to trap badgers in our* département, *it will be difficult*, *because there are two trapping companies that are squabbling*, *and this is a big problem*” (hunters’ federation, low-risk). However, relational constraints between categories were also identified, particularly between hunters and trappers: “*Hunting and trapping are different approaches*. *There are hunters who see trappers as competitors*, *so the trapper has to work discreetly*, *so his activities are not frowned upon*, *even though everyone has the same final goal*” (*lieutenant de louveterie*, high-risk).
- Coordination, an indispensable but time-consuming activity: The coordination and preparation of surveillance campaigns are essential, but time-consuming activities, particularly for active surveillance (meetings, preparation of the sampling kits for example): “*We have to keep repeating ourselves*. *Regular follow-up*, *which takes time*, *must be considered*” (hunters’ federation, high-risk). Some *lieutenants de louveterie* mentioned the complexity of their coordination activities, particularly in the context of regulatory constraints: “*It’s become a full-time job over the last few months*. *The workload has changed a lot and has been steadily increasing over the past decade*. *It gets a bit heavy-going sometimes and I struggle to get everything done […]*. *I feel a bit overwhelmed*” (*lieutenant de louveterie*, high-risk). The heavy workload of coordination activities was also felt by the veterinary officers in high-risk areas: “*Sylvatub is a lot of work*, *but the job isn’t just about administration*. *It’s about presence on the ground*. *There really is a human dimension*. *We lose a lot of time on coordination*, *but recognition is important and everyone now understands the value of their work*” (vet officer, high-risk).
- External factors: Some stakeholders did not wish to be involved in surveillance, although they did not give clear reasons as to why, or they spoke about factors external to the system, which veterinary officers could not easily managed. Furthermore, in December 2014, the Ministry of the Environment refused the request of a local hunting and wildlife commission to maintain the marten (*Martes martes*) on the list of pest species in a high-risk *département*. This provoked strong reactions, as the stakeholders did not understand this refusal and felt showed a lack of trust. The representatives of the hunting world then threatened to stop collaborating with veterinary services in the regulation of damage-causing species, and thus refused to participate in the Sylvatub system. This refusal to participate was made with the agreement of farmers’ representatives: “*Today we disengage from public orders*, *because there is not enough trust*. *However*, *we are not disengaging from our agricultural responsibilities*, *and if a farmer calls us we will go*” (hunters’ federation, high-risk). This refusal to participate in the program thus appears to have been a means of applying political pressure on the state, because the hunters are aware of their role and usefulness in the Sylvatub system.

### A recent network involving volunteers

- Voluntary actors: In most *départements*, hunters are considered by the coordinators to have reliable knowledge of a sufficiently high level for the detection of suspicious carcasses. However, the reporting of suspicions is not systematic and depends on various factors, such as the time available and the area (low- or high-risk), although "doubt" and "fear" were frequently mentioned: “*Even without training*, *hunters do not eat carcasses with lesions*. *If in doubt*, *some will throw them away*, *others will have them analyzed*. *Asking for a suspicious carcass to be analyzed is more a question of will and free time than a question of cost*” (farmer/hunter/trapper, high-risk). The fear of consequences in cases of infection seems to be higher in low-risk areas, potentially limiting the acceptability of surveillance: “*The problem in this* département *is that there are no bTB cases*, *so hunters are less inclined to make a declaration if they see something*, *for fear of the consequences*. *Hunters are afraid that their activities will be limited if they find something on a wild boar*. *In the affected areas*, *hunters are more aware and it is different”* (hunters’ federation, low-risk).
- A recently established network of stakeholders: Several stakeholders indicate that there is a historical gulf between the worlds of hunting and agriculture, which is continuing to widen and could hinder the effective functioning of the system and the establishment of good relationships between stakeholders. bTB is perceived a major source of concern, but also as a sensitive subject, particularly as concerns who is responsible for bTB cases: “*When we started looking for bTB in wild boars*, *everyone thought it meant that wild boars had bTB [laughing]*! *It's hard to reassure people afterwards*. *It’s not easy to explain what the research is*. *It creates a kind of psychosis*” (farmer/hunter/trapper, high-risk). Farmers expect hunters to play an active role in the fight against bTB in wildlife, but they are unaware of their missions and their constraints, which can cause tensions: “*The farmers make demands*. *They feel it is necessary to trap all the badgers*. *They ask hunters to do this directly*, *and some think that we have the right to trap the badger anyway but that we choose not to*. *They are not aware that we don’t have the right*, *and that we act only on requests from the authorities*” (*lieutenant de louveterie*, high-risk). The relationship between hunters and farmers is becoming increasingly difficult, particularly as fewer farmers are now hunting, which makes communication more difficult. Furthermore, the mobilization of farmers in surveillance activities is patchy and sometimes difficult. Some farmers become involved in surveillance by obtaining accreditation for trapping on the own land or by monitoring the traps placed by approved trappers (monitoring delegation). However, it is difficult to get some farmers involved, even as far as the surveillance of badgers on their own farms is concerned: “*It's hard to get people to trap*. *They are supposed to tell me where there are badgers*, *but they don’t*” (farmer/hunter/trapper, high-risk); “*If you find a diseased badger*, *the farmers must be controlled*. *It's a problem and a constraint*, *this herd control*: *it scares them*, *and that's why they don’t want to tell us if they see badgers*” (*lieutenant de louveterie*, high-risk).

Finally, the officers working for local veterinary services are in a difficult position within the network of stakeholders. They had little contact with hunters and hunting associations before the establishment of Sylvatub, which made it difficult to implement surveillance activities. Moreover, the veterinary services are considered to be the administrative authority, synonymous with regulation, constraints and controls, and hunters and their representatives therefore tend to distrust them: “*If you aren’t on the ground*, *it is difficult*. *Once you have human contact*, *it is OK*. *But they still see us as lecturing*, *coming to impose things*” (veterinary officer, high-risk); “*We have a bad image*: *we are in the administration*, *so we control*, *and they think we don’t know the environment*. *We start with a handicap in terms of communication*” (veterinary officer, high-risk).

### Determinant factors for the maintenance of awareness

- Recognition: A need for recognition, through compensation or information about their involvement in the surveillance system, was expressed by several stakeholders: “*It is important for trappers to tell farmers about their work (number of trappers*, *number of badgers trapped*, *kilometers traveled etc*.*)*, *because they underestimate the work of trappers and hunters*. *I think that could increase the loyalty and motivation of the trappers*” (*lieutenants de louveterie*, high-risk).
- More information and communication: A lack of information and communication about the surveillance system and its results was mentioned by many stakeholders involved in passive or active surveillance: “*What is missing*, *for the trappers and the hunting federation*, *is feedback*. *There are information meetings with partners at the end of surveillance campaigns*, *but no individual feedback for all trappers or hunters*. *Furthermore*, *the terms used in the letters (such as spoligotype*, *and prevalence rate) are too complicated for them; it’s not appropriate*” (hunters’ federation, high-risk). Some stakeholders even suggested setting up a more suitable, fun communication system. Finally, the stakeholders also called for wider communication with other categories, including farmers in particular, to present the importance of their involvement in the surveillance system.
- A greater involvement of farmers: The lack of involvement of farmers, who are in the front line in the fight against bTB in cattle, was often mentioned by stakeholders from the world of hunting, who expect farmers to do more: “*What I regret is that farmers aren’t aware enough; they don’t motivate themselves to do the training course in trapping*” (farmer/hunter/trapper, high-risk).
- Relational aspects as the key to good supervision, with local dynamism dependent on coordination: From the interviews, it seems that local coordinators play an essential role in maintaining the awareness and motivation of the collectors. This intermediate link between the administrative officers and field collectors is therefore essential. The collectors have more confidence in their local institution than in representatives of the administrative authority, who are generally seen in a poor light. The functioning of the network of shakeholders and a good relationship between categories of shakeholders seem to be fundamental, to ensure effective surveillance. Availability also seems to be an important expectation: “*There is a human dimension in the animation of this network*, *and people like it*. *I am convinced that you can have good coordination only if you are close to people; you have to take the time*. *There are the regulations*, *but there is also the human aspect behind*. *What is important is human presence and availability*” (veterinary officer, high-risk).
- Concern about the future: Some stakeholders expressed concern about the future of bTB surveillance and control, in both wildlife and cattle: “*It is an aging volunteer population and it’s unclear whether anyone will want to take over*, *given the many forms of leisure in current society*. *Some say that tuberculosis will still be around in 2035*, *but I don’t know who still be here then*. *It’s a long way off*” (veterinary officer, high-risk). Some stakeholders also expressed concern about changes to the Sylvatub system and its potential complexity: “*The management of bTB has got out of control*. *It is not easy to manage on the ground*. *I think it’s important that Sylvatub is a simple*, *fun system*, *given that it is entrusted to people in the field who are not scientists; we must never forget that*” (hunters’ federation, low-risk).

### How useful is the Sylvatub system?

Most of the participants did not call the usefulness of the Sylvatub system into question: “*I think Sylvatub is necessary and it is a good tool*” (hunters’ federation, low-risk); “*bTB surveillance in wildlife is really useful*, *too many farmers have been affected*” (hunter, high-risk); “*Before people had a tendency to destroy abnormal carcasses*, *but now some have the reflex to ask for analyses to improve knowledge*” (hunters’ federation, medium-risk); “*Sylvatub makes it possible to work on a more scientific basis*, *to have more structured work*. *I believe that this system has made it possible to target our research more accurately*” (veterinary officer, high-risk).

## Discussion

### Main results and recommendations

The evaluation of the Sylvatub system is of particular importance, as the persistence of infected wild populations may hinder the eradication of bTB from cattle in some countries [4]. Several authors have shown that surveillance notifications can be increased by developing an understanding of the reporting behavior of stakeholders and developing ways to influence that behavior in a positive manner [19]. However, these sociological aspects constitute major knowledge gaps in animal surveillance [7, 11]. It is, therefore, essential to assess the perception of surveillance by stakeholders and their willingness to participate in it, so as to limit under-detection and underreporting, particularly for passive surveillance [7, 18, 19], and to identify ways of improving surveillance [11, 13, 20, 21, 22].

This is, to our knowledge, the first study to explore the motivations of stakeholders to participate in bTB surveillance in wildlife in France. Thus, each opinion was qualitatively relevant and identified several important aspects to be taken into account in reflections on the levers that could be activated to increase dynamism and maintain the awareness of all stakeholders:

- The system seems to be perceived as useful, or even necessary, by all those interviewed. The concerns of the various stakeholders seem to be well-perceived and understood, which is important for the maintenance of their involvement in the long term. Thus, regular meetings with stakeholders involved in the surveillance system at the local, regional and national level must be continued to be organize to identify evolution in concerns and perceptions of the Sylvatub system;
- Many practical and regulatory constraints were mentioned, but many were partly offset by the belief that this surveillance was useful in the fight against bTB in wildlife. However, these constraints might demobilize stakeholders if their efforts are not sufficiently recognized by others or if the constraints outweigh the benefits obtained from surveillance (particularly as the volunteers could continue their leisure activities without the Sylvatub system). Thus, recognition through communication activities, compensation or in some other form, is fundamental to maintain participation in the system, particularly for volunteers [19]. If recognition appears to be insufficient at the long term to maintain stakeholders’ involvement, the actors’ network could be adapted, for example with the recruitment of professional trappers which could avoid the timely constraints;
- It is vital to improve communication and the provision of local information to stakeholders from the world of hunting, farmers and the public, despite the proposal of several training activities (theoretical and practical) since the introduction of the system, to bring hunters and farmers closer together. A communication system such as desired by the stakeholders already exists, in the form of a newsletter published regularly for the national partners, who should transfer it to their members. However, some of them do not received this newsletter as, which underlines a lack of communication at this level. This could easily be resolved by the enhancement of diffusion’s means of information. Furthermore, although the level of awareness can be increased by occasional information campaigns, it seems difficult to maintain a good level of awareness in the long term and sensitivity may therefore decrease with increasing surveillance time;
- Farmers, the main actors involved in the surveillance and control of bTB in cattle populations, are not sufficiently involved in these activities for wildlife. Information campaign about their utility in the fight against bTB and especially by trapping badger in their farm should be launched;
- Local coordination by *lieutenants of louveterie*, hunters’ federations and local veterinary services is a very time-consuming activity, but seems to be essential to ensure the involvement and participation of stakeholders and to maintain their motivation. The official recognition of their role will be important to ensure their implication at the long term.

We recruited stakeholders from only four *départements* for this study. We cannot, therefore, assume that the knowledge and perceptions of these stakeholders are representative of the national situation [18, 22]. However, in such qualitative approaches, the quality of the sample depends on the diversity of data collected rather than their representativeness [7, 15, 17, 20]. Nonetheless, participants from areas with all levels of risk were selected, and some had multiple roles (e.g. farmer, hunter, and trapper), making it possible for them to discuss several themes during the same interview and reducing the number of interviews required. Thus, we have conducted interviews until we have reached the theoretical saturation (no new information collected during interviews). Participants were selected according to their availability and willingness to participate in the study, which may have introduced a selection bias [10, 18, 22]. All interviews were conducted by the same person, due to time and cost restrictions. There may have been biases inherent to this interviewer, as he sometimes mentioned his links to the administration and spoke for long periods during the interview. However, despite the small number of participants and the need to interpret the results with care, these findings seem to shed light on the workings of the Sylvatub system, the perception of this surveillance system by stakeholders, and the factors driving and blocking their participation.

Thus, qualitative sociological approaches are useful tools for improving our understanding of the network of stakeholders, by taking into account their perceptions, needs and expectations. These approaches could increase the acceptability of evaluation and recommendations, through direct involvement of the stakeholders in the evaluation process [7, 22]. Semi-directive interviews have the advantage of being more flexible than closed questionnaires, making it possible to guide the discussion around themes previously defined according to the objectives of the study.

### Complementarity with other evaluation methods

This study was performed in addition to a quantitative assessment of the cost-effectiveness of surveillance, by assessing other attributes and trying to understand the rationale of certain political and operational decisions [10, 22]. In our scenario tree model, the likelihood of detecting TBLs depends on the awareness of hunters and the species hunted. Assumptions about disease awareness were based on the training and experience of the hunters (as a function of the risk in the *département*). The collection of dead or dying animals is also dependent on risk level, which affects the level of awareness of the partners in the field, and economic considerations (analyses are paid for by the hunter’s association in low-risk areas, but are reimbursed in medium- and high-risk areas). Thus, the small number of suspected cases reported in the Sylvatub system may reflect a poor acceptability of the surveillance measures or the negative consequences of reporting suspected cases of infection, consistent with the results of the evaluation by the Oasis flash method (limited acceptability, especially for the active surveillance on badgers) [12]. The use of semi-structured interviews has the advantage over Oasis evaluation of including more participants, including, in particular, people with different profiles active in the field (hunters, trappers, coordinators, officers, etc.), thereby providing a more comprehensive understanding of the surveillance system and making it possible to formulate context-dependent and more easily acceptable recommendations [13], through the development of a relationship of trust [12].

The various methods for evaluating surveillance systems may be considered complementary. For example, the quantitative evaluation showed that PSURV-SSC had a very good sensitivity [8], but the semi-quantitative Oasis evaluation identified heterogeneity in its local implementation [12]. The qualitative sociological evaluation highlighted a lack of acceptability, particularly for badger trapping. Thus, active surveillance appears to be effective, but the large sample sizes involved result in high costs [9] and relatively low acceptability, which may limit the sustainability of this SSC in the medium- and high-risk areas. The EC-SSC has a low individual sensitivity, partly offset by the large number of animals concerned [8]. Moreover, this SSC has a very high unit cost, due to the considerable coordination required [9], and its effective application seems to differ between *départements* [12], as a function of the level of awareness of stakeholders in the field. The improvement of the EC-SSC would therefore require a strengthening of coordination and local supervision activities for collectors. However, this would entail a significant cost, which might not lead to a marked increase in sensitivity in all areas, as the reporting of suspect cases is subject to behavioral and social factors influenced, in part, by the consequences of case detection (operational and economic consequences) and by the perception of the risk of bTB infection (lower in low-risk areas). Finally, the acceptability of the SAGIR-SSC seems to be good, probably because this network been in existence for many years. However, its effectiveness seems to be limited, both individually and collectively [8]. Moreover, increasing its sensitivity would not be easy as it is dependent on biological factors, such as the likelihood of an animal dying and being detected before its natural disappearance, which depends on the environmental context, food resources, and vegetation, for example.

Thus, even if a surveillance strategy has a theoretical advantage, such as low cost or high sensitivity, sociological approaches are essential to assess its acceptability, especially to collectors [18], with implications for improving communication, the management and the sustainability of the system [22]. However, motivations may differ between stakeholders (*e*.*g*. regulatory obligations, compensation for the public sector, competitive factors for the private sector). In such cases, multicriteria analysis is a useful tool for taking into account the different perceptions and interests of different groups of actors, which may conflict [23]. Several criteria could be considered in the decision-making process, to rationalize the choice of a surveillance strategy: direct economic impacts (costs of measures, potentially influencing state preferences), indirect economic impacts (export losses, which may influence the preferences of the agro-food industry or tourism sector), and social impacts (which may influence public opinion particularly in terms of mistrust for some control strategies involving total slaughter, for example) [24].

Moreover, although this study has helped to refine our understanding of the results obtained in semi-quantitative and quantitative evaluations, it is difficult to integrate its qualitative results into the data previously obtained. Indeed, there is a currently lack of gold standards in animal health for the interpretation of such qualitative results, and, thus, for deducing the acceptability of the Sylvatub system from quantitative results [7, 13, 18, 25].

A semi-quantitative method for assessing various acceptability criteria was recently developed [7], using participatory approaches and focus group discussions, which could easily be integrated into a multicriteria analysis to facilitate comparison with other evaluation attributes, such as economic or effectiveness attributes. This method can be used to study acceptability, by estimating the non-monetary benefits and costs of surveillance (incentives and disincentives that cannot be directly valued in monetary terms), the perceived economic value of health information [7, 26], the costs and benefits associated with the suspicion of infection and its declaration (positive and negative consequences), and the influence of social interactions between stakeholders on decision-making processes by visual methods (diagrams constructed by stakeholders). This qualitative information is then transformed into semi-quantitative information, by assigning scores according to the relative importance and impact of the decision to declare suspicions of infection [26]. This method provides a semi-quantitative tool, based on preferences and participatory approaches, to quantify incentives and disincentives that cannot be assessed directly in monetary terms [26], which could be considered a first step towards the development of a more standardized and reproducible approach to investigating acceptability, and linking such investigations to the evaluation of other attributes.

Thus, decision-making for disease surveillance and control in animal health depends not only on a scientific rationale based on epidemiological or economic criteria, but also on political and social factors [24]. It is vital to take into account the needs and interests of stakeholders involved in surveillance, to ensure the acceptability, sustainability and stability of the system and to ensure that the system provides adequate information [25]. The issues involved in this evaluation are thus technical, operational, and economic, but also institutional, societal or even political. The development of skills in sociology and economics, particularly in the context of the development of socioeconomic evaluations, would enrich existing methods of evaluation in animal health.

## Conclusion

The acceptability and operational feasibility of surveillance are essential for the correct functioning and effectiveness of medium- and long-term surveillance systems. Qualitative sociological approaches are thus essential, to analyze, perceive and understand the incentives and disincentives governing stakeholder participation in the surveillance system. The evaluation of a surveillance system should take into account socioeconomic and contextual factors that might influence decision-making, and should be dynamic, to detect changes in the behavior of stakeholders. However, there have been few studies on the interaction between resource allocation, cost-effectiveness, and the behavior of stakeholders, whereas feedback between surveillance and changes in disease levels may be influenced by contextual factors that alter the cost-effectiveness of the surveillance system [11].

Ensuring the sustainability of the Sylvatub system poses several fundamental challenges: improving our understanding of the epidemiological role of wild species in the bTB cycle, strengthen relational links between actors, improve the feel of trust between stakeholders and in the surveillance system. Our study, the first to assess stakeholders’ perceptions of the Sylvatub system since its beginning in 2011, allowed to investigate local perceptions of utility, concerns and acceptability of the surveillance activities and thus to identify key factors influencing the stakeholders’ willingness to participate to these activities at the long term. Thus, the major incentives are the feel of utility for the farmers, the recognition of the work by the other stakeholders of the Sylvatub system, the reinforcement of the communication about the surveillance activities and the results obtained, and the improvement of relationships with farmers, through their implication in the surveillance of bTB in wildlife. However, the sustainability of the system may be limited by the significant mobilization of human and financial resources in high-risk areas and by the time-consuming activities required, especially for coordination and trapping. The implementation of various forms of recognition and communication inside the stakeholders’ network could contribute to ensure their involvement in the system at long term.

## Supporting information

S1 File Main topics of the guide for interviews. (DOCX)

S1 Table List and characteristics of participants. (DOCX)
