# Supplementary material for: Perceptions and acceptability of some stakeholders about the bovine tuberculosis surveillance system for wildlife (Sylvatub) in France
Source: PLoS One. 2018 Mar 15;13(3):e0194447. doi: 10.1371/journal.pone.0194447 (PMC5854387; doi:10.1371/journal.pone.0194447)
Supplement: S1 File — (DOCX) [file pone.0194447.s001.docx]

**Supplementary Material S1 – Main topics of the guide for interviews**

**Introduction**

- Presentation of the study: context, objectives, main topics
- Presentation of the investigator: sociological study in the framework of a Masters II project
- Verbal consent, information about anonymization of the collected data, no good or bad response

**Topic 1: Description of the surveillance activities in relation to wildlife and the Sylvatub system**

*For all stakeholders: hunters and local hunting federations; trappers and “lieutenants de louveterie”; local federation of the national hunting and wildlife office; veterinary services*

● Activities relating to wildlife: which activities related to wild species are you involved in? Why? Since when?

● Activities relating to Sylvatub:

- Information about the existence and functioning of the Sylvatub system: Obtained from whom? When? How?

- Role in the surveillance system

- Relationships with other partners of the surveillance system

- Opinions and feelings: utility of this surveillance? Concerns? Benefits? Reasons for participating?

**Topic 2: Factors external to the Sylvatub system and local context**

*For all stakeholders, especially for the two* départements *at high-risk level in which external negative factors influence participation in the Sylvatub system.*

Some people and organizations are refusing to participate in surveillance this year: What is your position? Why?

**Topic 3: Factors internal to the Sylvatub system: incentives and disincentives**

*For all stakeholders: hunters and local hunting federations; trappers and “lieutenants de louveterie”; local federations of the national hunting and wildlife office; veterinary services*

● Brakes/disincentives:

- What constraints are there? Why?

- Technical aspects (material)

- Organizational aspects

- Relational aspects

- Financial aspects

- Workload

● Motivations/incentive factors:

- What are your motivations? Why?

- How could things be improved?

- What do you know about results of the surveillance?

- Perception of their utility? Recognition? (at local and national scale)

- What kind of recognition would you expect?

**Topic 4: Relationship between stakeholders and coordinators**

**●** Coordination**:**

*For all stakeholders: hunters and local hunting federations; trappers and “lieutenants de louveterie”; local federations of the national hunting and wildlife office; veterinary services*

- What kind of relations do you have with other stakeholders? When? How frequently?

- What information is transmitted between stakeholders?

- Relationships: sufficient? Quality? Difficulties?

- Knowledge about the network of stakeholders? Who should be contacted and when?

*For coordinators:*

- Coordination techniques: How do you coordinate the system?

- What difficulties are encountered in coordination activities?

- Needs?

- Stakeholder dynamics? Recruitment, maintenance of participation, facility of involvement etc.

● Training:

*For all stakeholders, but especially for hunters and local hunting federations*

- Training coordination

- Training participation

- Training quality? Sufficient for the role in the surveillance system?

- Needs?

**Topic 5: Functioning and utility of the Sylvatub system**

*For all stakeholders, but especially for state representatives (officers)*

- Observation pressure in the field?

- Homogeneity/heterogeneity between geographic areas?

- Opinion of the quality of surveillance (passive and active)?

- Ways and means of improving the quality of detection (passive surveillance)?

- Ways and means of improving the quality of declaration (passive surveillance)?

- Voluntary actors: weaknesses? What alternatives are there?

- Adjectives characterizing the Sylvatub system (5)?

- Expectations?

*For all stakeholders, but especially for hunters and local hunting federations*

- Number of suspected cases identified since the introduction of the Sylvatub system?

- Declarations of suspected cases: exhaustive? Reasons for non-declaration? [negative consequences, economic aspects, knowledge of procedures etc.]

**Conclusion**

General opinion concerning the Sylvatub system
