# Supplementary material for: Perceptions and acceptability of some stakeholders about the bovine tuberculosis surveillance system for wildlife (Sylvatub) in France
Source: PLoS One. 2018 Mar 15;13(3):e0194447. doi: 10.1371/journal.pone.0194447 (PMC5854387; doi:10.1371/journal.pone.0194447)
Supplement: S1 Table — (DOCX) [file pone.0194447.s002.docx]

**S2 Table – List and characteristics of participants**

|  | Collectors | | Coordinators | | Officers | | | | | |
| --- | --- | --- | --- | --- | --- | --- | --- | --- | --- | --- |
|  | Hunters | Trappers | Local hunting federations | “*Lieutenants de louveterie*” | State | ONCFS | | | | |
| **Low-risk level: 2 interviews** | | | | | | | | | |  |
| Stakeholder 1 | X |  | X |  |  |  | | | | |
| Stakeholder 2 | X | X |  |  |  |  | | | | |
| **Medium-risk level: 3 interviews** | | | | | | | | |  |  |
| Stakeholder 3 |  |  |  |  |  | X | | | | |
| Stakeholder 4 | X |  | X |  |  |  | | | | |
| Stakeholder 5 |  |  |  |  | X |  | | | | |
| **High-risk level (1): 7 interviews** | | | | | | | |  |  |  |
| Stakeholder 6 | X |  |  | X |  |  | | | | |
| Stakeholder 7 |  |  |  | X |  |  | | | | |
| Stakeholder 8 | X | X |  |  |  |  | | | | |
| Stakeholder 9 | X | X |  |  |  |  | | | | |
| Stakeholder 10 | X |  | X |  |  |  | | | | |
| Stakeholder 11 |  |  |  |  | X |  | | | | |
| Stakeholder 12 |  |  |  |  |  | X | | | | |
| **High-risk level (2): 8 interviews** | | | | | | |  |  |  |  |
| Stakeholder 13 |  | X |  |  |  |  | | | | |
| Stakeholder 14 | X |  | X |  |  |  | | | | |
| Stakeholder 15 | X |  |  | X |  |  | | | | |
| Stakeholder 16 | X | X |  | X |  |  | | | | |
| Stakeholder 17 | X |  |  |  |  |  | | | | |
| Stakeholder 18 | X | X |  | X |  |  | | | | |
| Stakeholder 19 | X |  |  |  |  |  | | | | |
| Stakeholder 20 |  |  |  |  | X |  | | | | |
